# Mutant *pfcrt *"SVMNT" haplotype and wild type *pfmdr1 *"N86" are endemic in *Plasmodium vivax *dominated areas of India under high chloroquine exposure

**DOI:** 10.1186/1475-2875-11-16

**Published:** 2012-01-11

**Authors:** Prashant K Mallick, Hema Joshi, Neena Valecha, Surya K Sharma, Alex Eapen, Rajendra M Bhatt, Harish C Srivastava, Patrick L Sutton, Aditya P Dash, Virendra K Bhasin

**Affiliations:** 1 National Institute of Malaria Research, Dwarka Sector-8, Delhi 110077, India; 2 National Institute of Malaria Research (Field Unit), Rourkela 769002, India; 3 National Institute of Malaria Research (Field Unit), Chennai 600037, India; 4 National Institute of Malaria Research (Field Unit), Raipur 492015, India; 5 National Institute of Malaria Research (Field Unit), Nadiad 387001, India; 6 Department of Biology, Center for Genomics & System Biology, New York University, New York, USA; 7 Department of Zoology, University of Delhi, Delhi 110007, India; 8 W.H.O SEARO, Delhi, India

## Abstract

**Background:**

Chloroquine resistance (CQR) phenotype in *Plasmodium falciparum *is associated with mutations in *pfcrt *and *pfmdr-1 *genes. Mutations at amino acid position 72-76 of *pfcrt *gene, here defined as *pfcrt *haplotype are associated with the geographic origin of chloroquine resistant parasite. Here, mutations at 72-76 and codon 220 of *pfcrt *gene and N86Y *pfmdr-1 *mutation were studied in blood samples collected across 11 field sites, inclusive of high and low *P. falciparum *prevalent areas in India. Any probable correlation between these mutations and clinical outcome of CQ treatment was also investigated.

**Methods:**

Finger pricked blood spotted on Whatman No.3 papers were collected from falciparum malaria patients of high and low *P. falciparum *prevalent areas. For *pfcrt *haplotype investigation, the parasite DNA was extracted from blood samples and used for PCR amplification, followed by partial sequencing of the *pfcrt *gene. For *pfmdr-1 *N86Y mutation, the PCR product was subjected to restriction digestion with AflIII endonuclease enzyme.

**Results:**

In 240 *P. falciparum *isolates with reported *in vivo *CQ therapeutic efficacy, the analysis of mutations in *pfcrt *gene shows that mutant SVMNT-S (67.50%) and CVIET-S (23.75%) occurred irrespective of clinical outcome and wild type CVMNK-A (7.91%) occurred only in adequate clinical and parasitological response samples. Of 287 *P. falciparum *isolates, SVMNTS 192 (66.89%) prevailed in all study sites and showed almost monomorphic existence (98.42% isolates) in low *P. falciparum *prevalent areas. However, CVIETS-S (19.51%) and CVMNK-A (11.84%) occurrence was limited to high *P. falciparum *prevalent areas. Investigation of *pfmdr-1 *N86Y mutation shows no correlation with clinical outcomes. The wild type N86 was prevalent in all the low *P. falciparum *prevalent areas (94.48%). However, mutant N86Y was comparably higher in numbers at the high *P. falciparum *prevalent areas (42.76%).

**Conclusions:**

The wild type *pfcrt *gene is linked to chloroquine sensitivity; however, presence of mutation cannot explain the therapeutic efficacy of CQ in the current scenario of chloroquine resistance. The monomorphic existence of mutant SVMNT haplotype, infer inbreeding and faster spread of CQR parasite in areas with higher *P. vivax *prevalance and chloroquine exposure, whereas, diversity is maintained in *pfcrt *gene at high *P. falciparum *prevalent areas.

## Background

Widespread *Plasmodium falciparum *chloroquine resistance (CQR) continues to pose a great challenge to malaria control efforts [1]. CQR is associated with single nucleotide polymorphisms (SNPs), which cause non-synonymous amino acid substitutions in the *P. falciparum *chloroquine resistance transporter (*pfcrt*) gene. As a result, the *pfcrt *gene is commonly used in epidemiological studies to characterize chloroquine treatment failure and ultimately monitor the emergence of drug resistance within endemic regions [2]. The replacement of a lysine (K) by a threonine (T) at codon 76 of the *pfcrt *gene seems to be a hallmark of CQR worldwide; both *in vitro *and *in vivo *tests suggest its use as an epidemiological tool for large scale studies of CQR in the field [3,4]. Twenty additional SNP's have been identified in the *pfcrt *gene from CQR field isolates, which may be due to selective pressure driving the population structure [5]. Two major haplotypes defined by specific mutations at amino acid positions 72-76 of *pfcrt*, CVIET and SVMNT, are associated with the geographic origin of CQR [6]. The CVIET haplotype is predominantly found in Southeast Asia and Africa, whereas the SVMNT haplotype is characteristic of South America, Papua New Guinea (PNG) [7], and the Philippines [8]. However, the CVIET haplotype has been observed in South America [9-11] and SVMNT haplotype in Southeast Asia [12]. Most African isolates share the CVIET haplotype of Southeast Asia, and it is hypothesized that this haplotype was imported through the Indian subcontinent [13]. Chloroquine-sensitive (CQS) strains are characterized by the CVMNK haplotype, irrespective of geographic origin. In India, the first *P. falciparum *chloroquine resistant case was reported in 1973 [14] and subsequently swept throughout the country [15]. The most prevalent *pfcrt *haplotype identified in India is SVMNT, although CVIET has also been observed [16-18]. A recent study reported that the SVMNT haplotype found in central India is closely related to the SVMNT genotype found in PNG [19], but it has yet to be determined if these two haplotypes have independent origins.

Similarly, SNPs in the *P. falciparum *multidrug resistance-1 (*pfmdr-1*) gene have been associated with reduced susceptibility to chloroquine treatment. Specifically, a SNP causing a nonsynonymous amino acid substitution at amino acid position 86, replaces the wild type asparagine (N) with a resistant tyrosine (Y) [20-22]. Most studies, including those performed in India, observe weak associations between *pfmdr-1 *mutations and parasite that are CQR [4,16,17,23].

Historically, *P. vivax *has been the principal malaria species in India; however, during the past two decades the incidence of *P. falciparum *has more than doubled; this has been attributed to a rise in chloroquine resistance across India [15]. Chloroquine resistance may have been exacerbated by misdiagnosis and consequently improper anti-malarial drug treatment, as chloroquine (CQ) is the first-line drug for vivax malaria in India [24]. Given that *P. falciparum *causes more severe and complicated forms of malaria, a resurgence of this parasite is alarming. Limited data is available on the distribution of *P. falciparum *resistant genotypes in regions typically dominated by *P. vivax*. Also, there is an overall lack of information on how these genetic haplotypes correlate with clinical resistance to CQ in India. Due to this, it is difficult to track CQR throughout India and nearly impossible to predict potential outbreaks of resistant parasites. This is a serious cause for concern, especially in regions of India where vivax malaria is the dominant species. During this study, CQR was monitored on molecular level by investigating the distribution of *pfcrt *haplotypes (concatenating mutations at codons 72-76 and codon 220) and the *pfmdr-1 *N86Y mutation across 11 field sites. Both high and low *P. falciparum *endemic sites in India were included to account for the impact of transmission intensity on the appearance of resistance and sensitive phenotypes with respect to these markers. Widespread parasite resistance to chloroquine was observed in India, while sensitive parasites appeared mostly in high *P. falciparum *endemic sites, like Chhattisgarh, Jharkhand, and Orissa [15].

## Methods

### Selection of isolates

The samples included in this study were collected from two independent studies: (i) 240 samples from a CQ therapeutic efficacy trial occurring in eight different field sites of varying *P. falciparum *prevalence (sites 1-8, below) and (ii) 47 samples collected by random malaria surveys in 3 additional sites (site 9-11, below). All of the samples were collected between 2002-2006. In the CQ therapeutic efficacy trial, patients were classified according to World Health Organization guidelines as: adequate clinical and parasitological response (ACPR), early treatment failure (ETF), and late treatment failure (LTF). All information about this clinical trial has been described elsewhere [25-27]. The Ethics Committee of National Institute of Malaria Research (NIMR) approved this study protocol. All individuals or the parents/guardians of children gave written informed consent before inclusion in the study.

### Study sites

**Figure 1:**
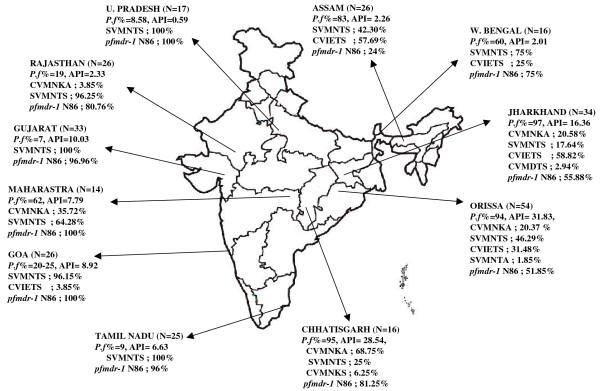
**Distribution of *pfcrt *haplotype and wild type *pfmdr-1 *N86**. Distribution of *pfcrt *haplotype and wild type *pfmdr-1 *N86, in different geographic areas of India with varied *P.falciparum *prevalence. *P.f *: *P.falciparum*, API: Annual parasite index, N: no. of isolates, *pfcrt *haplotype are CVMNKA,SVMNTS,CVIETS.

The proportion of *P. vivax *and *P. falciparum *varies in different parts of India. The study sites below represent the variable profiles of malaria transmission in India (Figure 1), classified into two groups according to *P falciparum *transmission. First, regions with high *P. falciparum *transmission: the Northeastern (Assam, W. Bengal), Eastern (Orissa, Jharkhand), Western (Maharastra), and Central (Chhattisgarh) regions of India. Second, regions with low *P. falciparum *transmission: North Central (UttarPradesh), Western (Rajasthan, Gujarat, Goa), and Southern (Tamil Nadu) part of India. These regions are described in detail below.

1. **Orissa, Eastern coastal state: **Sundergarh district (latitudes 21°35'N to 22°35'N, and longitude 83°32'E to 85°22'E) presents ideal ecological conditions for malaria, with undulating uplands intersected by forested hills, rocky streams, and paddy fields. This state contributes 23% to the country's malaria burden. In general, malaria endemicity ranges from meso- to hyperendemic malaria, with annual transmission. Sample collection from this field site occurred during year 2003. During this time, *P. falciparum *was the dominant infecting species, accounting for approximately 94% (n = 56331) of all recorded malaria infections.

2. **Assam, North-Eastern state: **Kamrup district (latitudes 25°46'N to 26°49'N, and longitudes 90°48'E to 91°50'E) is predominantly forest (1166.94 sq km of the district). Assam is near (80-100 km) the international borders of Myanmar, China and shares international borders with Bangladesh and Bhutan. The district had moderate and perennial malaria transmission with approximately 83% (n = 4645) due to *P. falciparum *of all malaria cases recorded in year 2002.

3. **Jharkhand, Eastern state: **Simdega district (latitudes 20°10'N to 20°40'N, and longitudes 84°E to 84°34'E) covered with forested area (1194.50 sq km of the district) is highly endemic for *P. falciparum *malaria. A total of 97% (n = 8,618) of the recorded malaria cases in year 2006 were *P. falciparum *infected, and it represented approximately 7% of the country's total malaria burden in same year.

4. **West Bengal, Eastern state: **Darjleeng district (latitude 27°42'N and longitude 88°16'E) is covered with forested hills, as it is located in the foothills of Himalayas. The district shares an international border with Nepal, Bangladesh, and Bhutan. Malaria transmission in the district is moderate, with seasonal malaria transmission of malaria. During the sample collection year 2003, *P. falciparum *comprised approximately 60% (n = 2157) of malaria infections in this region.

5. **Goa, Western coast: **Panaji (latitudes 15°N and longitude 73°E), in the North Goa district, is an urban area surrounded by plains. It is a tourist destination with much ongoing construction work, which provides a higher rate of human migration and promotes a niche for malaria transmission. *P. vivax *is the predominant malaria species in Panaji, however the percentage of *P. falciparum *malaria has increased up to 20-25% in last 5 years. The field site record indicates that *P. falciparum *comprised 20.5% (n = 1298) of all the malaria cases in the year 2004.

6. **Gujarat, Western state: **Anand district (latitude 22°57'N and longitude 72°93'E) is a rural area with plains. Anand is prone to malaria epidemics, but typically has low and seasonal transmission. *P. vivax *is the predominant malaria species in this district; only 7% (n = 1358) of the malaria was attributed to *P. falciparum *infection in the year 2005..

7. **Rajasthan, Western state: **Udaipur district (latitude 24°35'N and longitude 73°41'E) is located in the plains and has low and seasonal transmission. In year 2003, *P. falciparum *contributed only 19% (n = 1151) of the total malaria burden within this district.

8. **Tamil Nadu, Southern coastal state: **Rameswarum Island (latitude 9°22'N and longitude 78°52'E) in Ramanathapuram district of Tamil Nadu is a pilgrimage site, where human travel from various parts of country is a daily routine. Rameswarum Island is an endemic region for *P. vivax *malaria and has perennial transmission. Samples were collected in year2004, and only 9% (n = 359) of the malaria burden was attributed to *P. falciparum *in same year.

9. **Chhattisgarh, Central state: **Kanker district, (latitudes 20°6'N to 20°24'N, and longitudes 80°48'E to 81°48'E) have small hilly pockets throughout the district. Malaria endemicity is high, with annual transmission. In sample collection year 2006, *P. falciparum *malaria accounted for approximately 95% (n = 20573) of the total malaria burden in the same region.

10. **Maharastra, Western state: **Gadchiroli district (latitude 20°6'N and longitude 80°E) in the state of Maharastra is covered with dense forest and hills. The forest covers approximately 75.9% of the geographical areas of the district. Malaria endemicity is high, with annual transmission. In year 2002, *P. falciparum *accounted for approximately 62% (n = 4732) of the malaria burden in the district.

11. **Uttar Pradesh, North Central State: **Gautam Budh Nagar district (latitude 28°34'N and longitude 77°33'E) is an industrial area, located in the plains, heavily populated with migrant labourers from across the country. Malaria endemicity is low and seasonal, with *P. vivax *accounting for the highest burden of malaria within this region. In year 2002, *P. falciparum *only accounted for approximately 9% (n = 17) of the total malaria cases recorded in the district.

### Genomic DNA isolation and PCR amplification

Genomic DNA was extracted from blood spots collected on Whatman No.3 filter paper, using a QIAamp DNA Blood Mini Kit (Qiagen, Hilden, Germany).

*pfcrt *and *pfmdr-1 *genes were amplified from extracted DNA by nested PCR assays. Primary and nested PCR assays were performed in a 20 μl volume reaction with 0.75 U of Taq DNA polymerase, 2.5 mM MgCl2, 0.25 mM dNTP's and 0.01 mM of primers. The primary PCR product was diluted 1:10 times, before performing the nested reaction. All the primer sequence and PCR conditions are described in Table 1. For *pfmdr-1 *N86Y mutation, the nested PCR product was subjected to restriction digestion at 37°C with 5U of AflIII (MBI, Fermentas) endonuclease for 6 h.

**Table 1:** Description of primer sequences and respective PCR amplification conditions

Amplified region	Primer sequence #	Amplification condition
*pfcrt gene (codons 32-119) *	Primary 20: PFCRT 1F 5'CCGTTAATAATAAATACAGGC-3' PFCRT 1R 5'CTTTAAAAATGGAAGGGTGT 3' Nested 16: PFK76T 5'-5'GGCTCACGTTTAGGTGGA3' PRK76T 5'TGAATTTCCCTTTTTATTTCCAAA 3'	94°C 5 min; 94°C 30 sec, 56°C 1 min, 60°C 90 sec (40X), 60°C 3 min. 94°C 5 min; 94°C 30 sec, 52°C 45 sec, 72°C 45 sec sec (35X), 72°C 5 min.

*pfcrt gene (codons 181-222) *	Primary 20: PFCRT 1F 5'CCGTTAATAATAAATACAGGC-3' PFCRT 1R 5'CTTTAAAAATGGAAGGGTGT 3' Nested 48: PFA220S 5'CTTATACAATTATCTCGGAGCAG3' PRA220S 5'ATAATAAAAACAAAGTTTAAGTGT 3'	Same as for above primary 94°C 5 min; 94°C 30 sec, 51°C 45 sec, 72°C 45 sec sec (35X), 72°C 5 min.

*pfmdr-1 gene (N86Y) *	Primary 16: MDR1F 5'ATGGGTAAAGAGCAGAAAGA3' MDR1R 5'AACGCAAGTAATACATAAAGTCA3' Nested 47: MDR1FN 5'AGATGGTAACCTCAGTATCA3' MDR1RN 5'TTACATCCATACAATAACTTG3'	94°C 5 min; 94°C 30 sec, 54°C 45 sec, 72°C 45 sec sec (35Χ), 72°C 5 min. 94°C 5 min; 94°C 30 sec, 49°C 45 sec, 72°C 45 sec sec (35X), 72°C 5 min.

^# ^Primer sequences were adapted from Dorsey et al. [20]; Vathsala et al. [16]; Jelinek et al. [28] and Lim et al. [29]

### Sequence analysis

Final *pfcrt *PCR product was purified with a column based gel extraction kit (MDI, India), and then was subjected to automated sequencing. The sequences obtained were aligned using MegAlign module of Lasergene Version 5 (DNASTAR, Inc., USA).

### Statistical analysis

A *χ*^2 ^test or Fisher's exact test (GraphPad Software, Inc) was used to compare the frequency of *pfcrt *haplotypes between groups of high and low *P. falciparum *endemicity.

## Results

The data in this study has been analysed in two ways: (i) regional distribution of mutations (sites 1-11) and (ii) mutation analysis in relation to *in vivo *CQ efficacy clinical trial (sites 1-8).

### Regional distribution of *pfcrt *and *pfmdr-1 *mutations

After the *pfcrt *gene was sequenced for all 287 isolates from 11 different study sites, the mutations were assembled into a single haplotype by concatenating the amino acids located in positions 72-76 and position 220. The distribution of *pfcrt *haplotypes is presented in Table 2. The mutant SVMNT-S haplotype was the most prevalent haplotype within all sites, comprising 192 (66.89%) of all the isolates observed in this study. Not surprisingly, there was less diversity in the low transmission regions, compared to the high transmission regions; however, this limited diversity was dominated by the mutant SVMNT-S haplotype (125 of 127 isolates or 98.42%). There were three major haplotypes found within regions of high transmission across India: (i) the mutant haplotype SVMNT-S comprises 67 (41.87%) of the total; (ii) the mutant haplotype CVIET-S comprises 56 (35.00%) of the total; and (iii) the wild type haplotype CVMNK-A comprises 34 (21.25%) of the total. Compared with the low transmission regions, both the CVIET-S mutant and the wild type CVMNK-A haplotype occurred at significantly higher frequencies in the high transmission regions (P < 0.001, *χ*^2^).

**Table 2:** Distribution of *pfcrt *haplotypes, *pfmdr-1 *N86Y mutation and (*pfcrt-pfmdr-1*) 2 loci mutation i.e. combined genotype

	no. of isolates (%) in high *P.falciparum *prevalent areas							no. of isolates (%) in low P.falciparum prevalent areas					
***pfcrt haplotype***^***a***^	**Assam**	**Orissa**	**Jharkhand**	**West Bengal**	**Chhattisgarh**	**Maharastra**	***total***	**Gujarat**	**Goa**	**Tamil Nadu**	**Rajasthan**	**Uttar Pradesh**	***total***

CVMNK-A	0	11(20.37)	7(20.58)	0	11(68.75)	5(35.72)	34(21.25)	0	0	0	1(3.85)	0	1(0.78)
CVIET-S	15(57.69)	17(31.48)	20(58.82)	4(25)	0	0	56 (35)	0	1(3.85)	0	0	0	1(0.78)
SVMNT-S	11(42.30)	25(46.29)	6(17.64)	12(75)	4(25)	9(64.28)	67(41.87)	33(100)	25(96.15)	25(100)	25(96.25)	17(100)	125(98.42)
CVMDT-S	0	0	1(2.94)	0	0	0	1(0.62)	0	0	0	0	0	0
CVMNK-S	0	0	0	0	1(6.25)	0	1(0.62)	0	0	0	0	0	0
SVMNT-A	0	1(1.85)	0	0	0	0	1(0.62)	0	0	0	0	0	0
***pfmdr mutation***^*b*^													
N86 *wt*	6(24)	28(51.85)	18(52.94)	12(75)	13(81.25)	14(100)	91(57.23)	32(96.96)	26(100)	24(96)	21(80.76)	17(100)	120(94.48)
N86Y *mt*	19(76)	26(48.14)	16(47.05)	4(25)	3(18.75)	0	68(42.76)	1(3.03)		1(4)	5(19.23)		7(5.51)
***Combined genotype***^***a***^													
CVIETS -N	3(12)	8(14.81)	11(32.35)	0	0	0	22(13.83)	0	1(3.85)	0	0	0	1(0.78)
CVIETS -Y	12(48)	9(16.66)	9(26.47)	4(25)	0	0	34(21.38)	0	0	0	0	0	0
SVMNTA -N	0	1(1.8)	0	0	0	0	1(0.62)	0	0	0	0	0	0
SVMNTS -N	3(12)	10(18.51)	1(2.9)	12(75)	1(6.25)	9(64.28)	36(22.64)	32(96.96)	25(96.15)	24(96)	20(76.92)	17(100)	118(92.91)
SVMNTS-Y	7(28)	15(27.77)	5(14.7)	0	3(18.75)	0	30(18.86)	1(3.03)	0	1(4)	5(19.23)	0	7(5.51)
CVMNKA-N	0	9(16.66)	7(20.58)	0	11(68.75)	5(35.71)	32(20.12)	0	0	0	1(3.85)	0	1(0.78)
CVMNKA-Y	0	2(3.7)	0	0	0	0	2(1.25)	0	0	0	0	0	0
CVMDTS -Y	0	0	1(2.9)	0	0	0	1(0.62)	0	0	0	0	0	0
CVMNKS -N	0	0	0	0	1(6.25)	0	1(0.62)	0	0	0	0	0	0

^a^Mutated amino acid is underlined, ^b ^*wt *Wild type, *mt *Mutant type

For the 286 isolates tested for N86Y mutation throughout India, the wild type N86 was observed in 211 (73.77%) isolates. Regional distribution of *pfmdr-1 *mutation is presented in Table 2. The wild type N86 was significantly more prevalent in the low transmission regions (n = 120, 94.48%), whereas in high transmission regions, the wild type (N86) and the mutant (N86Y) were found in similar proportions, 91 (57.23%) and 68 (42.76%) respectively (P < 0.0001, *χ*^2^). The N86Y was predominantly observed in Assam (76.00%) and Orissa (51.85%) isolates, whereas wild type N86 was observed more in isolates from West Bengal (75.00%), Chhattisgarh (81.25%) and Maharastra (100%).

### Distribution of haplotypes

The combination of *pfcrt *haplotypes and *Pfmdr-1 *mutations revealed nine haplotypes distributed at variying rates within different field sites (Table 2). The haplotype SVMNTS-N occurred in 154 (53.84%) isolates and was detected in all sites. Its significant occurrence in low *P. falciparum *transmission areas 118 (92.91%) isolates is understandable, as a higher number of SVMNT-S and N86 was previously observed in these areas. The other mutant haplotype, SVMNTS-Y was observed in 5.51% isolates from low *P. falciparum *transmission areas, while CVIETS-N and CVMNKA-N each were observed only one time. In contrast, there was considerable variation observed in the haplotypes from isolates collected in high *P. falciparum *transmission areas i.e. 32 (20.12%) wild type CVMNKA-N and various mutant types SVMNTS-N (n = 36, 22.64%), CVIETS-Y (n = 34,21.38%), SVMNTS-Y (n = 30,18.86%), CVIETS-N (n = 22,13.83%), CVMNKA-Y (n = 2, 1.25%) and one isolate (0.62%) of the following haplotypes SVMNTA-N, CVMDTS-Y, CVMNKS-N.

### Distribution of *pfcrt *and *pfmdr-1 *mutations in relation to *in vivo *CQ efficacy

A total of 240/287 isolates collected from the CQ therapeutic efficacy study [25-27] were sequenced for analysis of *pfcrt *gene. The mutant K76T was observed irrespective of treatment outcome. Of the 106 isolates identified as ACPR (i.e. CQ sensitive isolates), 87 (82.07%) were found to be mutant K76T, while only 19 (17.92%) contained the wild *pfcrt *haplotype CVMNKA. The mutant K76T was observed in the remaining 134 cases classified as treatment failures (i.e. ETF and LTF). Though the wild type was observed infrequently across India, it was almost exclusively detected in high transmission regions (n = 18,94.73%) compared to low transmission region (n = 1,5.26%). The mutant *pfcrt *haplotype SVMNT-S was prevalent in all treatment outcomes, i.e. observed in 71 (66.98%) ACPR, 15 (60.00%) ETF, and 76 (69.72%) LTF isolates. On the other hand, occurrence of mutant haplotype CVIET-S was almost entirely limited to high *P. falciparum *transmission regions and was distributed in all the clinical outcomes, 15 (14.15%) ACPR, 10 (40.00%) ETF, and 32 (29.35%) LTF isolates. Only a single isolate from Goa, a low transmission region was identified by the CVIET-S haplotype. Instances of rare haplotypes were also observed; CVMDT-S haplotype was observed in one of the LTF isolate from Jharkhand and SVMNT-A haplotype was observed in one of the ACPR isolates from Orissa. The distribution of *pfcrt *haplotypes in various clinical outcomes is presented in Table 3.

**Table 3:** The distribution of mutations in different clinical responses

CQ RESPONSE		ASSAM^a^	ORISSA	JHARKHAND	W.BENGAL	GUJARAT	GOA	TAMIL NADU	RAJASTHAN	TOTAL
**ACPR**	***pfcrt haplotype***^***b***^									
	CVMNK-A	0	11(45.83)	7(41.17)	0	0	0	0	1(3.85)	19(17.92)
	CVIET-S	5(41.66)	3(12.50)	6(35.29)	1(14.28)	0	0	0	0	15(14.15)
	SVMNT-A	0	1(4.16)	0	0	0	0	0	0	1(0.94)
	SVMNT-S	7(58.33)	9(37.50)	4(23.52)	6(85.71)	6(100)	8(100)	6(100)	25(96.25)	71(66.98)
	***pfmdr *mutation^c^**									
	N86 wt	1(9.09)	18(75.00)	13(76.47)	6(85.71)	6(100)	8(100)	6(100)	21(80.76)	79(75.23)
	N86Y mt	10(90.90)	6(25.00)	4(23.52)	1(14.28)	0	0	0	5(19.23)	26(24.76)
	***Combined genotype***^***b***^									
	CVMNKA N	0	9(37.50)	7(41.17)	0	0	0	0	1(3.85)	17(16.19)
	CVMNKA Y	0	2(8.33)	0	0	0	0	0	0	2(1.90)
	SVMNTS N	0	6(25.00)	1(5.88)	6(85.71)	6(100)	8(100)	6(100)	20(76.92)	53(50.47)
	SVMNTSY	6(54.54)	3(12.50)	3(17.64)	0	0	0	0	5(19.23)	17(16.19)
	SVMNTA N	0	1(4.16)	0	0	0	0	0	0	1(0.95)
	CVIETS N	1(9.09)	2(8.33)	5(29.41)	0	0	0	0	0	8(7.61)
	CVIETSY	4(36.36)	1(4.16)	1(5.88)	1(14.28)	0	0	0	0	7(6.66)

**ETF**	***pfcrt haplotype***									

	CVIET-S	2(40)	4(66.66)	2(100)	1(50)	0	1(11.11)	0	0	10(40.00)
	SVMNT-S	3(60)	2(33.33)	0	1(50)	1(100)	8(88.88)	0	0	15(60.00)
	***pfmdr mutation***									
	N86 wt	4(80)	2(33.33)	1(50)	1(50)	1(100)	9(100)	0	0	18(72.00)
	N86Y mt	1(20)	4(66.66)	1(50)	1(50)	0	0	0	0	7(28.00)
	***combined genotype***									
	SVMNTS N	2(40)	1(16.66)	0	1(50)	1(100)	8(88.88)	0	0	13(52.00)
	SVMNTSY	1(20)	1(16.66)	0	0	0	0	0	0	2(8.00)
	CVIETS N	2(40)	1(16.66)	1(50)	0	0	1(11.11)	0	0	5(20.00)
	CVIETSY	0	3(50.00)	1(50)	1(50)	0	0	0	0	5(20.00)

**LTF**	***pfcrt haplotype***									

	CVIET-S	8(88.88)	10(41.66)	12(80)	2(28.57)	0	0	0	0	32(29.35)
	SVMNT-S	1(11.11)	14(58.33)	2(13.33)	5(71.42)	26(100)	9(100)	19(100)	0	76(69.72)
	CVMDT-S	0	0	1(6.66)	0	0	0	0	0	1(0.91)
	***pfmdr mutation***									
	N86 wt	1(11.11)	8(33.33)	4(26.66)	5(71.42)	25(96.15)	9(100)	18(94.73)	0	70(64.22)
	N86Y mt	8(88.88)	16(66.66)	11(73.33)	2(28.57)	1(3.84)	0	1(5.26)	0	39(35.77)
	***combined genotype***									
	SVMNTS N	1(11.11)	3(12.50)	0	5(71.42)	25(96.15)	9(100)	18(94.73)	0	61(55.96)
	SVMNTSY	0	11(45.83)	2(13.33)	0	1(3.84)	0	1(5.26)	0	15(13.76)
	CVIETS N	0	5(20.83)	5(33.33)	0	0	0	0	0	10(9.17)
	CVIETSY	8(88.88)	5(20.83)	7(46.66)	2(28.57)	0	0	0	0	22(20.18)
	CVMDTSY	0	0	1(6.66)	0	0	0	0	0	1(0.91)

^a ^Respective percentage values in brackets, ^b ^Mutated amino acid is underlined, ^c ^*wt *Wild type, *mt *Mutant type

In total, 239 isolates underwent successful PCR amplification and endonuclease digestion for *pfmdr-1 *N86Y mutation. The distribution of the *pfmdr-1 *mutation N86Y in the different clinical outcomes is shown in Table 3. In brief, both the wild type and the mutant alleles were detected in each of the three different clinical outcomes; no significant association was found between wildtype and mutant alleles and the treatment outcome. The wild type N86 allele was observed in 167 (69.87%) of the 239 isolates and distributed accordingly; 79 (75.23%) ACPR, 18 (72.00%) ETF, and 70 (64.22%) as LTF. Similarly mutant N86Y allele was found in 26 (24.76%) of isolates classified as ACPR, 7(28.00%) as ETF and 39 (35.77%) as LTF. The combination of *pfcrt *haplotypes and *Pfmdr-1 *mutations revealed again, that SVMNTS-N prevail in all the category of clinical outcome also; 53 (50.47%) isolates in ACPR, 13 (52.00%) isolates in ETF, 61 (55.96%) isolates in LTF.

## Discussion

In this study, the distribution of *pfcrt *haplotypes and the *pfmdr-1 *mutation (N86Y) has been described across 11 sites in India and evaluated for a correlation between the prevalence of mutations and the clinical outcome of CQ treatment. The distribution of *pfcrt *haplotypes in association with the clinical outcome of treatment supports that the K76T mutation is the most predictive marker of CQR in the field [3,4]. The wild type CVMNK-A haplotype was found to be exclusively restricted to the ACPR outcome group (P < 0.001, Fisher's exact test). However, the wild type CVMNK-A haplotype was not the predominant haplotype in the ACPR outcome group; in fact, 82.07% of the isolates identified as ACPR carried a mutant haplotype. From this data, it is proposed that the typing of molecular markers for CQ resistance may infer an intrinsic characteristic of the parasite, but may not necessarily be sufficient to predict treatment outcome. Treatment outcome may depend on other factors, such as host-parasite or host-drug interaction [30]. Exposure-related host immunity may play an essential role in naturally clearing the parasite infection, irrespective of their response to any drug. Some studies have shown that host immunity is also associated with clearance of resistant genotypes [20,31].

Additionally, the mutant haplotype SVMNT-S (characteristic of South American or PNG CQR isolates) was observed in all sites across India, regardless of the clinical outcome. Observation made in this study support earlier reports regarding the prevalence of SVMNT-S haplotype among the CQR isolates in India [16-18]. Perhaps the most striking observation observation in the study is that the SVMNT-S haplotype is endemic across all the sites of low *P. falciparum *malaria transmission, while in high transmission regions there are multiple mutant *pfcrt *haplotypes observed. These different haplotype combinations observed in regions of high transmission might be indicative of random mating patterns among parasites within these regions, which could be influenced by the local rate of malaria transmission. The role of transmission in the evolution and spread of drug resistance remains a matter of debate, as it has been hypothesized by some that low transmission may decrease circulating drug resistance, while others hypothesize that low transmission may increase drug resistance [32,33]. These studies signify the effect of transmission on altering the spread of drug resistance. In this study, the distribution of mutations hints that the spread of CQR might be under the influence of the transmission intensity and the distribution of anti-malarial drugs. Though complexity of infection (minimum number of clones within an infection) was not characterized in this study, it is common to observe a higher proportion of multiclonal infections in regions of high transmission, compared to regions of low transmission [34-37]. The ubiquitous nature of mutant SVMNT-S *pfcrt *haplotype in the low transmission regions in this study may be a direct consequence of higher inbreeding potential of resistant parasites due to low frequencies of complex and multiclonal infections. An inbreeding population, consisting mostly of resistant genotypes could spread parasite drug resistance expeditiously [32,38]. This expansion of mutant SVMNT-S is likely to be enhanced by extensive exposure of CQ in these *P. vivax *predominated areas, as chloroquine is the first-line drug for vivax malaria in India. A similar expansions hasbeen observed for the *pfmdr-1 *wild type N86 in these *P. vivax *prevalent areas. Again inbreeding might be the cause for this expansion in low transmission regions.

The significant presence of wild type CVMNK-A in high *P. falciparum *prevalent areas (Orissa, Jharkhand, Chhattisgarh) supports earlier reports on acquisition of immunity in high endemic areas, which creates a natural ecological refuge for drug sensitive parasites [31,39]. This could possibly explain the occurrence of the wild type haplotype in high *P. falciparum *prevalent areas. Further, appearance of the mutant haplotype CVIET-S(characteristic of Southeast Asian CQR parasite) is expected to be observed within eastern and northeastern parts of the country, because this region shares international borders with Bangladesh, Nepal, Bhutan, Myanmar and China and harbour migrant populations. The CVIET-S haplotype might be indicative of high drug pressure in regions of high transmission, as earlier observations have shown that the CVIET-S haplotype have higher IC_50 _for CQ [17,40]. Similarly, the prevalence of *pfmdr-1 *mutant N86Y found in these areas may be involved in modulating the CQ response in Indian isolates. Isolates with the two loci mutation (*pfcrt-pfmdr-1*) were limited to high *P. falciparum *transmission regions, where N86Y appeared with both the *pfcrt *mutant haplotype SVMNT-S and CVIET-S. These two mutant haplotypes were observed in high numbers at Assam, Orissa, and Jharkhand, whereas Chhattisgarh shows a higher frequency of the wild type for both loci. This study has not evaluated IC_50 _values for particular *pfcrt *haplotypes, but the observed prevalence of the mutant SVMNT-S, even in the high transmission regions may be an indication of increased transmission potential of these resistant parasites [40]. The use of amodiaquine as monotherapy or in combination has been associated with the prevalence of SVMNT haplotype in chloroquine resistant areas [41,42]. In India, amodiaquine was used as presumptive anti-malarial drug for CQR parasite in 1980's in these high transmission regions [43-45] and may be a possible explanation for the introduction and spread of SVMNT-S haplotype. On the other hand, a recent investigation from central India, using multilocus microsatellites, compared the evolutionary proximity of Indian SVMNT-S haplotype with other parts of the world and reveals its close relation to the SVMNT genotype found in PNG [19]. So, overall the spread of SVMNT is intriguing and require further investigation about its origin and spread in India.

The observed prevalence of the wild type *pfmdr-1 *N86 allele could be a cause for concern, because a recent study reported that the selection of this allele is associated with resistance to one of the artemisinin-based combination therapy (ACT-Coartem^®^) desseminated in East Africa [46]. This allele has also been associated with a decreased sensitivity to lumefantrine *in vitro *[47,48]. Furthermore, the significant prevalence (P < 0.001) of wild type N86 in low *P. falciparum *prevalent areas, seems to be fixed and may result in an easy escape for parasites exposed to this drug combination. Ultimately, this fixation could lead to the rapid spread of resistant parasites. However, a recent investigation of therapeutic efficacy of Coartem^® ^in the high *P. falciparum *transmission regions marks successful outcome without any selection of N86 [49].

## Conclusions

This studyobserved a striking pattern in fixation of mutant SVMNT *pfcrt *haplotype at low *P. falciparum *prevalent areas, which raise concerns about faster spread of anti-malarial resistance in these areas. The absence of wild type *pfcrt *haplotype in most part of the country may lead to a situation where no reversal of wild type would happen even in the absence of CQ pressure. This study leads towards understanding the role of malaria transmission intensity in spread of anti-malarial resistant parasite in India, and it will be useful in designing anti-malarial treatment policy.

## Competing interests

The authors declare that they have no competing interests.

## Authors' contributions

PKM carried out the experiment design, experimental work, data analysis, and manuscript writing. NV, APD, HJ conceived and coordinated the study. NV, SKS, AE, RMB, HCS, APD supervised all the field work. PLS has contributed in data analysis and manuscript writing. VKB supervised overall work and contributed in data analysis and manuscript writing. All authors read and approved the final manuscript.
